# 3-Pentadecylphenol (PDP) as a Novel Compatibilizer for Simultaneous Toughened and Reinforced PA10,12 Composites

**DOI:** 10.3390/polym16131915

**Published:** 2024-07-04

**Authors:** Yuwei Jin, Qi Zhang, Xiaokun Zhai, Hao Teng, Youmei Du, Jing Lu, Sumaiya Farzana, Patrick C. Lee, Ruiyan Zhang, Faliang Luo

**Affiliations:** 1 State Key Laboratory of High-Efficiency Utilization of Coal and Green Chemical Engineering, Ningxia University, Yinchuan 750021, China; jyuweil0@163.com (Y.J.);; 2 School of Chemistry and Chemical Engineering, Ningxia University, Yinchuan 750021, China; 3 Chuanghe New Material Technology Jiangsu Co., Ltd., Yangzhou 225000, China; 4 Key Laboratory of Photovoltaic Materials, School of Materials and New Energy, Ningxia University, Yinchuan 750021, China; 15908981813@163.com; 5 Multifunctional Composites Manufacturing Laboratory (MCML), Department of Mechanical and Industrial Engineering, University of Toronto, Toronto, ON M5S 3G8, Canadapatricklee@mie.utoronto.ca (P.C.L.)

**Keywords:** polyamide, toughness, POE, notch impact strength

## Abstract

The utilization of polyamide 10,12 (PA10,12) composites in various industries has been limited constrained by their inherent low toughness, making it a challenge to achieve a balance between toughness and structural integrity through conventional elastomer addition strategies. Herein, we introduce a straightforward method for the concurrent toughening and reinforcement of PA10,12 composites. This is accomplished by blending polyolefin elastomer (POE) and 3-pentadecylphenol (PDP) with the PA10,12 matrix. The incorporation of 5 wt% PDP effectively blurred the PA10,12/POE interface due to PDP’s role as a compatibilizer. This phenomenon is attributed to the formation of intermolecular hydrogen bonds, as evidenced by Fourier Transform Infrared Spectroscopy (FTIR) analysis. Further investigation, using differential scanning calorimetry (DSC), elucidated the crystallization thermodynamics and kinetics of the resulting binary PA10,12/POE and ternary PA10,12/POE/PDP composites. Notably, the crystallization temperature (*T_c_*) was observed to decrease from 163.1 °C in the binary composite to 161.5 °C upon the addition of PDP. Increasing the PDP content to 10% led to a further reduction in *T_c_* to 159.5 °C due to PDP’s capacity to slow down crystallization. Consequently, the ternary composite of PA10,12/POE/PDP (92/3/5 wt%) demonstrated a synergistic improvement in mechanical properties, with an elongation at break of 579% and a notch impact strength of 61.54 kJ/m^2^. This represents an approximately eightfold increase over the impact strength of unmodified PA10,12. Therefore, our work provides the potential of PDP as a compatibilizer to develop nylon composites with enhanced stiffness and toughness.

## 1. Introduction

Polyamide (PA10,12) is extensively used across the automobile, electrical, and machinery industries due to its superior electrical insulation, mechanical properties, minimal friction, and favorable processing characteristics [1]. However, its relatively low toughness has been a significant barrier to its broader application and further development. A traditional strategy to toughen enhance the toughness of PA10,12 is through the incorporation of elastomers. Polyolefin elastomer (POE) is a type of thermoplastic elastomer showing potential in various applications, including the manufacturing of wires, cables, everyday essentials, which is attributed to POE’s high elasticity, outstanding aging resistance, and robust chemical stability [2,3,4]. Moreover, the unique microstructure of POE, which includes crystalline regions serving as the physical cross-links and the non-polar octene chains contributing to its softness, endows it with exceptional elasticity [5,6]. When POE is dispersed within a PA10,12 matrix, it can effectively dissipate external forces and enhance impact stress. Under increased impact stress, the POE phase leads to facilitates the formation of numerous craze streaks and shear bands from points of stress concentration, which, in turn, significantly enhance the toughness of the PA composite. For example, the development of superior tough PA/POE-g-MAH composites has been reported [7].

However, the enhancement in toughness is often accompanied with a reduction of tensile and flexural strength. While the incorporation of elastomers can typically increase the impact strength of polyamide [8,9,10], it often leads to a notable reduction in tensile strength. This decline results from the phase separation, which contributes to inadequate compatibility between polar polyamide and non-polar elastomer [11,12,13]. Consequently, enhancing interfacial compatibility is crucial for optimizing the overall performance of the composite material. Liu et al. [4] demonstrated that blending various ethylene-octene copolymers (POE) with high-density polyethylene (HDPE) could be influenced by the molecular weight of the POE, affecting the extent of toughening achieved. To ensure a uniform dispersion of elastomers and improve their compatibility within the polyamide matrix, Ding et al. [14] utilized polyolefin elastomer grafted with maleic anhydride (POE-g-MAH) as a compatibilizer via blending PA10,12 with HDPE resins. This approach resulted in a significant enhancement in notch impact strength, which could reach as high as 600 J/m^2^ with a POE-g-MAH content of 40%. Additionally, the introduction of POE-g-MAH led to a marked improvement in the interfacial properties of the PA10,12/HDPE blend. Zeng et al. [15] reported the effects of grafting maleic anhydride onto ethylene-propylene-diene monomer (EPDM) to create a PA6/EPDM-g-MAH blend. This modification resulted in a substantial increase in the impact strength of the PA composite, which was 64.8% higher at ambient temperature and 106.6% higher at low temperatures compared to unmodified PA6.

3-pentadecylphenol (PDP), characterized by its phenolic microstructure and 15 alkyl chains, has been effectively utilized to modify the properties of polyamide composites. In our prior research [16], the incorporation of 30 wt% PDP was incorporated to tune the intermolecular hydrogen bonds between PA6,12 and PDP. This strategic modification led to a significant enhancement in the impact strength of the PA composite, which increased significantly to 17.5 kJ/m^2^. However, the improvement in toughness was accompanied by a substantial reduction in tensile strength, dropping down to 32 MPa. Due to the unique chemical structure of PDP, we further explored its potential as a novel compatibilizer. By regulating the intermolecular hydrogen bonding between PA and multi-walled carbon nanotubes (MWCNTs), we developed a terpolymer composite of PA10,12/PDP/MWCNTs. This composite exhibited not only high stiffness but also superior toughness. The impact strength of this advanced composite, impressively, increased from 8 kJ/m^2^ to 76 kJ/m^2^, showcasing the significant potential of PDP in the development of high-performance polyamide-based materials.

Herein, we introduce a straightforward approach to the development of a reinforced and toughened PA10,12 composite through the integration of polyolefin elastomer (POE) and 3-pentadecylphenol (PDP). The addition of 5% PDP to the PA10,12/POE blend results in a notable blurring of the interface between the two materials due to the reduction in the dispersed phase size of POE. This observation suggests an enhanced compatibility between the phases. The establishment of intermolecular hydrogen-bonding interactions between PA10,12 and PDP was confirmed using Fourier-transform infrared spectroscopy (FTIR), providing molecular-level insight into the improved interaction. Additionally, scanning electron microscopy (SEM) was employed to characterize the morphological changes in the composites, both with and without the addition of PDP as a compatibilizer. Subsequently, the crystallization kinetics of the composites were analyzed using differential scanning calorimetry (DSC), offering a deeper understanding of the thermal behavior. Ultimately, mechanical testing was conducted to assess the stiffness and toughness of the developed nanocomposites. The ternary composite, with a formulation of PA10,12/POE/PDP at weight percentages of 92/3/5, demonstrated a remarkable enhancement in both toughness and strength, showing the efficacy of the PDP compatibilizer in achieving a synergistic improvement in material properties.

## 2. Experimental Section

### 2.1. Materials

In the present investigation, we utilized a commercially available of PA10,12 (the grade name of B150) with a density of 1.03 g/cm^−1^ was purchased from Shandong Guangyin New Material Co., Ltd. (Zibo, China). Additionally, we procured 15 alkyl phenol (PDP) (M_W_ = 304 g/mol, purity > 90%) was purchased from Shanghai TCI Co., Ltd. (Shanghai, China) Furthermore, Polyolefin elastomer (POE) with a grade of LC565 was provided by LG company (Chungcheongnam-do, Republic of Korea), with MFI of 5 (190 °C, 2.16 kg), glass transition temperature of −54 °C.

### 2.2. Sample Preparation

**Figure 1:**
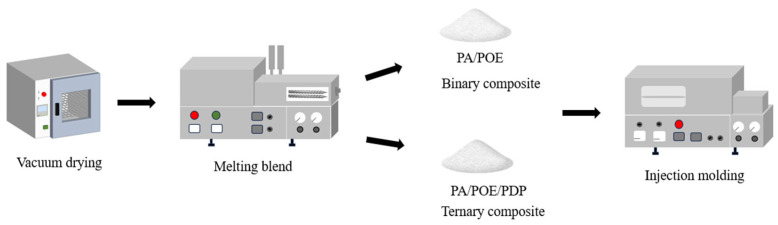
The schematic of preparation process for ternary PA10,12/PDP/POE composites.

The ternary composite consisting of PA10,12, polyolefin elastomer (POE), and 3-pentadecylphenol (PDP) was fabricated via a melt mixing approach, followed by extrusion and injection molding. To begin, both PA10,12 and POE pellets were pre-dried under vacuum conditions at a temperature of 85 °C for a duration of 12 h. Concurrently, PDP powder was also dried in a vacuum at a milder temperature of 40 °C for the same period of 12 h. Post-drying, the blend of PA10,12/POE/PDP was extruded using a micro-twin screw extruder operated at a controlled screw rotation speed of 30 revolutions per minute (RPM). The extrusion process involved a temperature gradient, with the extruder temperatures set at 160 °C, 200 °C, 215 °C, and 220 °C from the feed barrel to the extruder head. Following extrusion, the composite material was injection molded into standard spline molds. This was achieved using a micro-injection molding technique with specific mold dimensions of 80 × 10 × 4 mm^3^ and 75 × 5 × 2 mm^3^. The injection molding parameters were optimized as follows: an injection temperature of 220 °C, a mold temperature of 45 °C, an injection time of 10 s, and a clamping time of 30 s, all applied at a constant pressure of 0.6 MPa [16]. A schematic representation of the composite preparation process is depicted in Figure 1. The specific binary and ternary mixture ratios are shown in the Table 1 below.

### 2.3. Characterizations

Microscopic analysis was conducted to evaluate the microstructural characteristics of the composite samples. Specifically, scanning electron microscopy (SEM) was utilized, employing a German Zeiss Sigma 300 instrument operated at an accelerating voltage of 10 kV. To prepare the samples for SEM observation, they were first fractured to reveal the cross-sectional morphology. Subsequently, the fractured surfaces were sputter-coated with a thin layer of gold to enhance conductivity and resolution, with the coating process conducted under an inert argon atmosphere to prevent oxidation. In addition to SEM, atomic force microscopy (AFM) was also employed to further investigate the surface topography and to construct a phase diagram of the composite material. The AFM measurements were performed using a Bruker Dimension AFM system, with a scan size of 10 μm to capture detailed surface features and phase distributions.

The chemical composition and molecular interactions within the neat PA10,12, binary blends, and ternary composites were examined using Fourier transform infrared (FTIR) spectroscopy. The FTIR spectra were acquired at room temperature with a Spectrum Two instrument, scanning a wavenumber range from 4000 to 400 cm^−1^ at a spectral resolution of 0.5 cm^−1^. This range encompasses key vibrational modes that provide insights into the molecular structure and intermolecular bonding within the samples.

X-ray diffraction (XRD) analysis was conducted to delineate the crystalline structure of the samples. The measurements spanned a range of angles from 5° to 50°, which is conventional for identifying crystallographic phases and assessing unit cell dimensions. The XRD was equipped with a D8 Advance attachment, and the analysis was performed under the following parameters: a tube voltage of 40 kV and a current of 40 mA, with all measurements taken at room temperature. These conditions provided a robust dataset for the structural characterization of the materials.

The non-isothermal crystallization kinetics of all composite samples were meticulously assessed using a TAQ20 differential scanning calorimeter (DSC) from TA Instruments, USA, Delaware. The analysis commenced with heating the samples to a temperature of 220 °C, where they were maintained for a period of 5 min to erase any prior thermal history. This step is critical to ensure that each sample begins the experiment with a uniform thermal state. Subsequently, the samples underwent controlled cooling to a temperature of 30 °C, employing a range of cooling rates varying from 5 °C to 40 °C per minute. This multi-rate cooling regimen facilitates the study of crystallization kinetics over a broad spectrum of conditions. Finally, the samples were reheated to 220 °C to gather essential enthalpy data, which are pivotal for understanding the thermal transitions and crystallization behavior of the materials.

Throughout the cooling process, the relative crystallinity, represented as *X*(*t*), is defined as the ratio of enthalpy produced during cooling process to the total enthalpy release:

The function relationship between the relative crystallinity *X*(*t*) and crystallization time of the non-isothermal crystallization kinetics of PA10,12 and PA10,12/20% PDP/1.5% carbon nanotubes (CNTs) composites can be defined by the following formula [17]:

(1) $$X(t)=\frac{\int_{t_{0}}^{t}\frac{dH_{c}}{dt}dt}{\int_{t_{0}}^{t_{\infty }}\frac{dH_{c}}{dt}dt}$$

where *t*_0_ and *t* represent the start and end time of polymer crystallization, respectively, and *H_c_* is the enthalpy of crystallization during the cooling process.

In this study, the non-isothermal crystallization parameters of PA10,12 and their composites were obtained by means of Mozhisen method [18]. MoZhishen method is a Formula (4) based on Avrami equation (Formula (2)) and Ozawa equation (Formula (3)), which is about establishing the relationship between *β* and *t*.

(2) $$X\left(t\right)=1-\exp\;(-Kt^{n})$$

(3) $$1-X\left(t\right)=\exp\;\left[-\frac{F(T)}{\beta^{n}}\right]$$

In the above formula, *X*(*t*) is relative crystallinity; *β* is the cooling rate; *F*(*T*) is the temperature function.

(4) lnβ=logF(T)−αlnt

Dumbbell-shaped samples, measuring 75 × 5 × 2 mm^3^, were tested for tensile properties using an electronic universal material testing machine (Model GTM8050S) at a strain rate of 10 mm/min. For the Izod Impact test, samples with dimensions of 80 × 10 × 4 mm^3^ were allowed to equilibrate for 24 h. Subsequently, the notch depth of the samples was precisely set to 2 mm using a notching tool (JJ-TEST, China, HeBei). The notched impact tests were then performed on an electronic combined pendulum impact testing machine (Model XJC-25ZD, China, HeBei) at a temperature of 25 °C. The test was conducted in accordance with the Izod impact test method, with the impact energy and speed being set at 2.75 J and 3.5 m/s, respectively.

## 3. Results and Discussion

**Figure 2:**
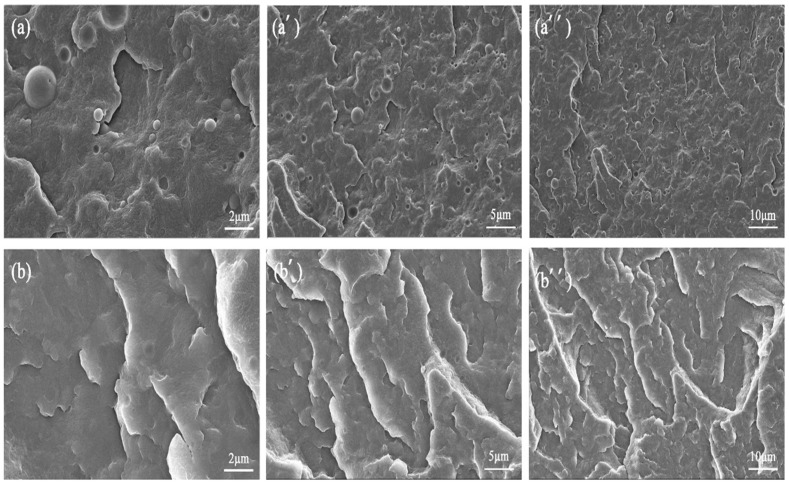
SEM photos of binary PA10,12/POE composites (**a**–**a″**) and trinary PA10,12/POE/PDP composites (**b**–**b″**).

The dispersion of elastomeric components is crucial to the mechanical properties of the PA10,12/POE/PDP composites. To observe the microstructure of PA10,12/POE and PA10,12/PDP/POE composites, SEM images are presented in Figure 2. When the POE content is 3% (Figure 2a–a″), the POE phases exhibit a typical sea-island phase structure, with sharp boundaries for the POE clearly visible. With the addition of 5% PDP, the interface of PA10,12/POE gradually became blurred (Figure 2b–b″), indicating an improvement in the thermodynamic compatibility between the two phases. Therefore, PDP is expected to act as a compatibilizer, enhancing the PA10,12/POE interface. The XRD patterns of PA10, PA12, and their composite materials were observed using X-ray diffraction to assess the impact of POE and PDP on the crystalline morphology of PA10 and PA12, as shown in Figure S1.

**Figure 3:**
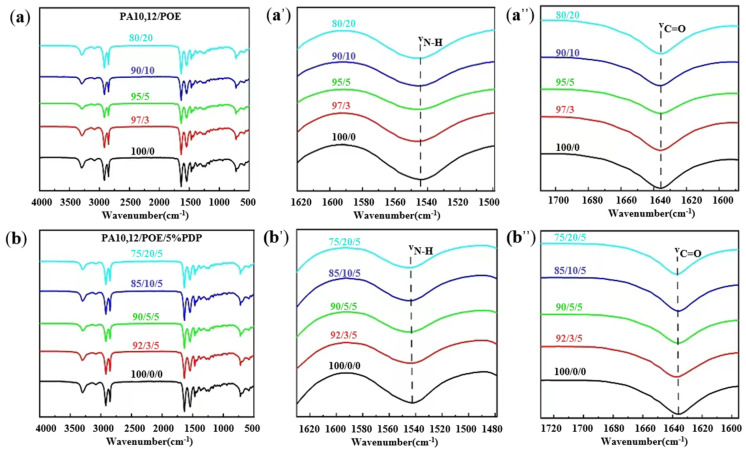
(**a**,**b**) Full spectrum of FTIR spectra of PA10,12 and its composites in the wave number region of 4000–500 cm^−1^ (**a**′) 1500~1620 cm^−1^ for N-H bending vibration (**a″**) 1600~1700 cm^−1^ for C=O tensile vibration. (**b**′) 1480~1630 cm^−1^ for N-H bending vibration (**b″**) 1600~1735 cm^−1^ for C=O tensile vibration.

To discuss the role of PDP compatibilizer in PA10,12/POE/PDP ternary composites, the hydrogen bond interactions between PDP and PA10,12 are revealed by FTIR. Figure 3a,b display the infrared spectra of PA10,12 and its composites within the wavenumber range of 4000 to 500 cm^−1^. The pristine PA10,12 exhibits characteristic absorption peaks at 1542, 1635, 2849, 2917, and 3300 cm^−1^, which are attributed to the N-H bending vibration, C=O stretching vibration, symmetric and asymmetric methylene stretching vibrations, and N-H stretching vibration, respectively [19,20,21]. The wavenumbers corresponding to the C=O stretching vibration and the N-H bending vibration remain consistent upon the incorporation of POE (Figure 3b′,b″), demonstrating that no H-bonds are established between PA10,12 and POE. After the addition of 5% PDP, the wavenumber of C=O stretching vibration increases from 1637 to 1638 cm^−1^, while the peak value of N-H stretching vibration adjusts from 1543 cm^−1^ to 1542 cm^−1^, indicating the establishment of H-bonds between the hydrogen atom (N-H) in PA10,12 and the oxygen atom of PDP. The intermolecular H-bonds were also established in our previous investigation [16].

**Figure 4:**
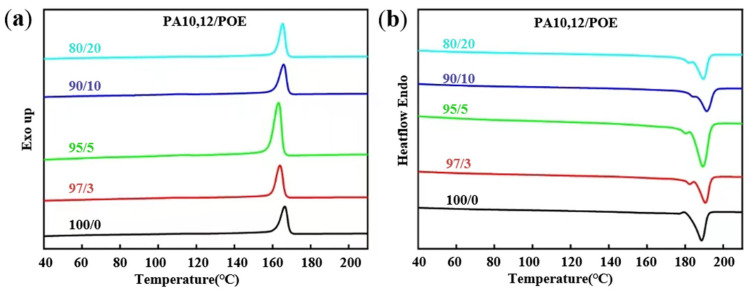

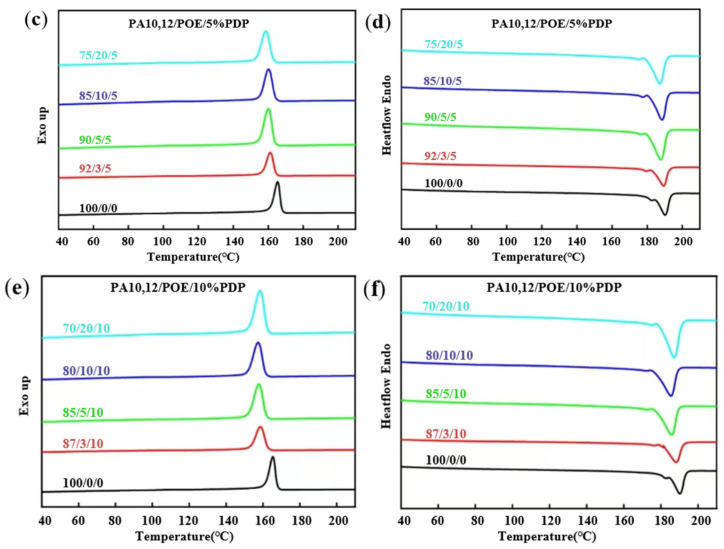
(**a**,**b**) Crystallization and melting parameters of PA10,12. (**c**,**d**) Crystallization and melting parameters of PA10,12/POE/5%PDP composites. (**e**,**f**) Crystallization and melting parameters of PA10,12/POE/10%PDP composites.

Crystalline structures play a critical role on the mechanical performance of composites. The crystallization and melting curves of neat PA10,12, PA10,12/POE (97/3), PA10,12/POE/PDP (92/3/5), and PA10,12/POE/PDP (87/3/10) composites are shown in Figure 4. The purpose of the data shown in the Figure 4 is to more clearly explain the role of POE and PDP on the crystallization of PA10,12. The corresponding parameters are systematically listed in Table 2. The crystallization temperature of the PA10,12/POE (95/5) blend is reduced from 166.5 °C to 163.2 °C, and the melting temperature is lowered from 192.3 °C to 189.4 °C compared to pure PA10,12. It indicated that the crystalline fragilized and crystalline lattice became thinner. And, combined with half crystallization time (*t*_1/2_), it obviously increased from 0.15 min to 0.38 min while incorporation of POE and PDP molecules, which demonstrated that POE and PDP molecules restricted PA crystalline growth. With the addition of 5% PDP, the T_c_ of all PA10,12/POE/PDP composites shifted to lower temperature of 161.1 °C. With the addition of 5% PDP, the *T_c_* of all PA10,12/POE/PDP composites shifted to lower temperature of 161.1 °C.

Analysis of the composite melting curve reveals that the melting temperature (*T_m_*) of the PA10,12/POE composite decreases with the addition of POE. Moreover, with addition of 5% PDP, the *T_m_* of the composite material also shifts in the low temperature direction, being 2.9 °C lower than that of neat PA10,12, which may be contributed from the intermolecular hydrogen bonds between PA and PDP molecules [16]. According to Scherrer equation [22], the lamellar thickness depends on the *T_m_*, with the lower *T_m_* resulting in thinner PA crystals. Consequently, the incorporation of PDP molecules could lead to thinner PA-crystals [23,24].

**Figure 5:**
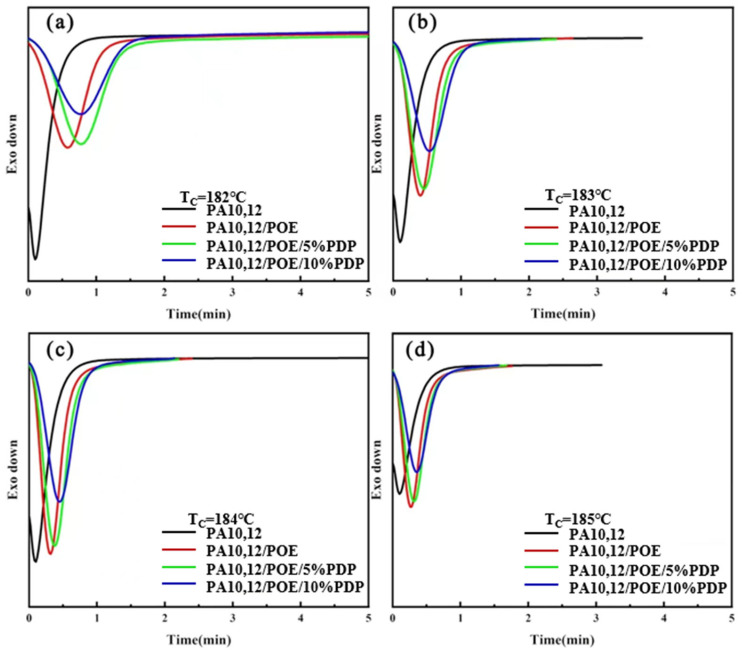
(**a**) Isothermal crystallization curves of PA10,12, PA10,12/POE and PA10,12/POE/PDP composites at 182 °C. (**b**) Isothermal crystallization curves of PA10,12, PA10,12/POE and PA10,12/POE/PDP composites at 183 °C. (**c**) Isothermal crystallization curves of PA10,12, PA10,12/POE and PA10,12/POE/PDP composites at 184 °C. (**d**) Isothermal crystallization curves of PA10,12, PA10,12/POE and PA10,12/POE/PDP composites at 185 °C.

**Figure 6:**
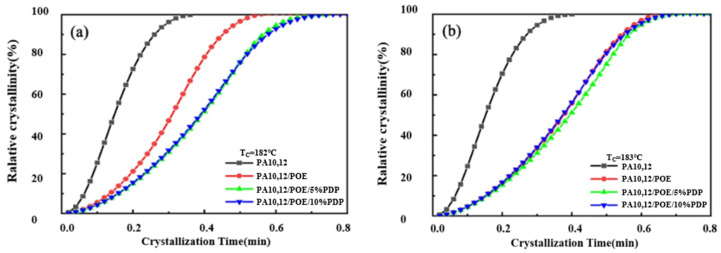

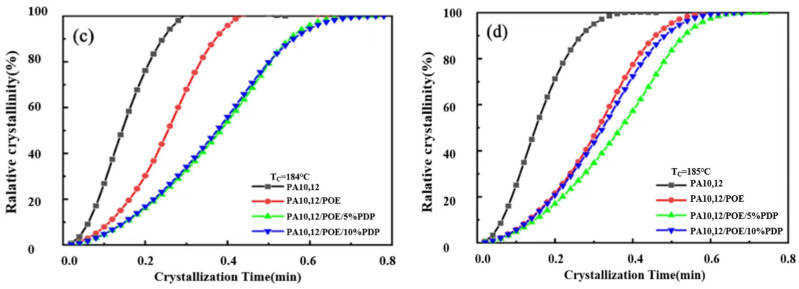
(**a**) The relationship between relative crystallinity and time of PA10,12 and its composites at isothermal temperature of 182 °C. (**b**) The relationship between relative crystallinity and time of PA10,12 and its composites at isothermal temperature of 183 °C. (**c**) The relationship between relative crystallinity and time of PA10,12 and its composites at isothermal temperature of 184 °C. (**d**) The relationship between relative crystallinity and time of PA10,12 and its composites at isothermal temperature of 185 °C.

**Figure 7:**
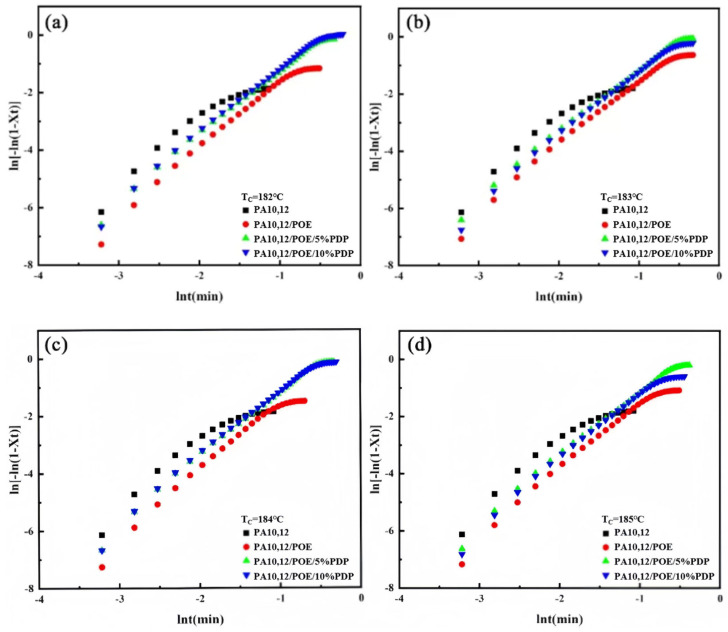
(**a**) Avrami analysis of isothermal crystallization of PA10,12 and PA10,12/POE/PDP composites at 182 °C. (**b**) Avrami analysis of isothermal crystallization of PA10,12 and PA10,12/POE/PDP composites at 183 °C. (**c**) Avrami analysis of isothermal crystallization of PA10,12 and PA10,12/POE/PDP composites at 184 °C. (**d**) Avrami analysis of isothermal crystallization of PA10,12 and PA10,12/POE/PDP composites at 185 °C.

Figure 5 depicts the isothermal crystallization curves of PA10,12, PA10,12/POE, PA10,12/POE/PDP composites at various isothermal crystallization temperatures: 182 °C, 183 °C, 184 °C and 185 °C, respectively. As the isothermal crystallization temperature increases, a notable trend emerges across all composites: the crystallization peak progressively shifts to longer times, and the peak broadens. This phenomenon signifies a deceleration in the crystallization rate with rising temperature. At a constant crystallization temperature, the half crystallization time (*t*_1/2_) of PA10,12/POE (97/3) is 0.31 min longer than that of neat PA10,12 (0.15 min), resulting from the crystal growth restriction imposed by POE molecules. After integrating the crystallization curve, the relationship between relative crystallinity and time is depicted in Figure 6. All composite materials exhibit an “S” shaped crystallization curve, with half crystallization time, and *t*_1/2_ of the composite significantly increasing with the incorporation of PDP at all the isothermal crystallization temperatures, consistent with the literature findings [25]. This demonstrates that PDP molecules further slowdown the crystalline rate of PA10,12, which might be contributed to the intermolecular H-bonding interactions. To study the kinetics of isothermal crystallization, we employed the Avrami equation [26] to correlate the relationship between the relative degree of crystallinity (*X_t_*) and time (*t*). This analysis is graphically represented in Figure 7, offering insights into the crystallization behavior of the materials under study. The Avrami index (*n*) and the crystallization rate constant (*k*) within the relative crystallinity range of 10% to 75%, and their specific values were shown in Table 3. The Avrami index (*n*), which is indicative of the crystallization mechanism, increased from 1.9 for the neat PA10,12 to 2.3 for the PA10,12/POE composite. This increase is attributed to the heterogeneous nucleation effect of POE, which serves as a secondary phase within the composite. However, the further addition of PDP did not result in a further increase in the Avrami index (*n*), suggesting that PDP does not significantly alter the nucleation and growth mechanism of PA10,12. In terms of the crystallization rate, the rate constant (*k*) for the neat PA10,12 exhibited a trend of decreasing values with an increase in crystallization temperature. Interestingly, the value of *k* increased from 0.79 for the neat PA10,12 to 1.45 for the PA10,12/PDP/POE (93/5/3) composite. This suggests that the incorporation of POE into the PA10,12 matrix tends to decrease the overall crystallization rate, potentially due to the disruption it causes in the polymer chain mobility and crystallization kinetics. The addition of POE results into the considerable increase of PA crystallinity due to the heterogeneous nucleation, which can be supported by an increased value of *n* (Avrami Index) from 1.9 to 2.4.

Furthermore, the half crystallization time (*t*_1/2_), a key parameter in characterizing the kinetics of isothermal crystallization, is also detailed in Table 3. To illustrate, at a crystallization temperature of 182 °C, the *t*_1/2_ of the neat PA10,12 was observed to be 0.15 min. This value increased to 0.31 min for the PA10,12/POE composite. The increasement of *t*_1/2_ in the composite can be attributed to the crystallization restriction imposed by the POE molecules, which act to hinder the crystallization process. With addition of 5% PDP, the *t*_1/2_ obviously further increased to 0.4 min, possibly attributed to the well-dispersed POE phase. Moreover, with a further increase in PDP content to 10%, *t*_1/2_ increased to 0.73 min due to the intermolecular H-bonds between PDP and PA10,12 matrix.

**Figure 8:**
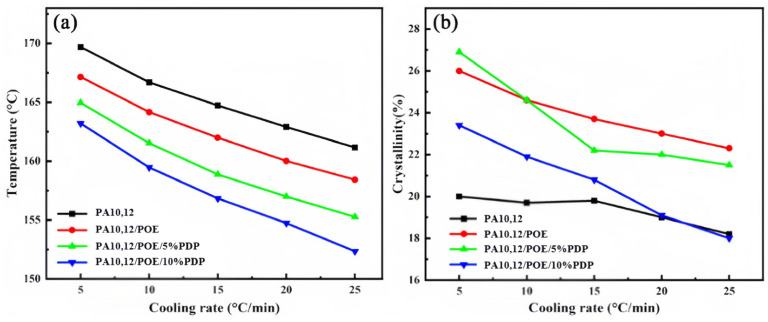
Crystallization temperature of pure PA10,12, binary PA10,12/POE and ternary PA10,12/POE/PDP composites at different cooling rates from 5 to 25 °C/min (**a**) Crystallinity curve (**b**).

To explore the kinetics of non-isothermal crystallization, we examined the initial crystallization temperature and the crystallization peak temperature for PA10,12, PA10,12/POE, and PA10,12/POE/PDP composites across a range of cooling rates, as illustrated in Figure 8. It was observed that the crystallization temperature (*T_c_*) systematically decreased with an increase in the cooling rate, spanning from 5 °C/min to 25 °C/min. For instance, at a cooling rate of 10 °C/min, the crystallization temperature (*T_c_*) for the binary PA10,12/POE composite exhibited a reduction from 166.7 °C to 164.2 °C. The non-isothermal crystallization curves of PA10, 12, PA10,12/POE, and PA10,12/POE/PDP composites are shown in Figure S2.

**Figure 9:**
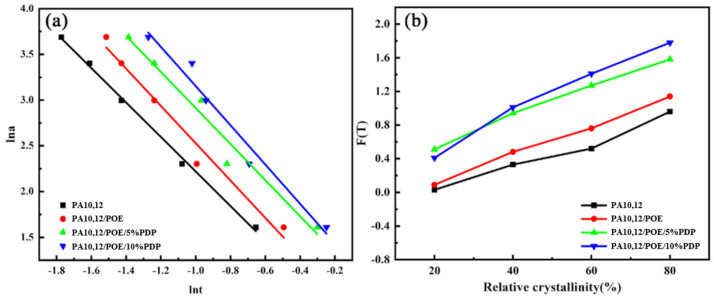
The *T_c_* was determined for pure PA10,12, binary blends of PA10,12 with POE, and ternary composites of PA10,12 with both POE and PDP at cooling rates ranging from 5 to 25 °C per minute; (**a**) *lna* vs. *lnt* at 50% at degree of crystallinity of 50%; (**b**) The function F(T) at various levels of relative crystallinity can be determined using the *Mo* equation.

According to the “*Mo*” method calculation [19], the specific kinetic parameters for the non-isothermal crystallization process have been determined in Table 4. The slope and intercept of the fitted linear curves of −α and lnF (T) values can be obtained through the “*Mo*” equation, as illustrated in Figure 9. For a certain degree of crystallinity, taking 40% as an example, the F(T) of PA10,12/POE/PDP in the ternary system increased from 0.73 to 1.01 with the increasement of PDP content, a greater increase compared to the composite without PDP. This further indicates that the polymer formed by PDP and POE after the addition of PDP hinders the growth of PA10,12 crystal nuclei. The α values of PA10,12, PA10,12/POE, PA10,12/POE/PDP range from 1.33 to 1.59, 1.92 to 2.23, and 1.91 to 2.16, respectively, indicating that the addition of POE and PDP does not alter the nucleation mode of PA10,12 consistent with the previous conclusions [27].

**Figure 10:**
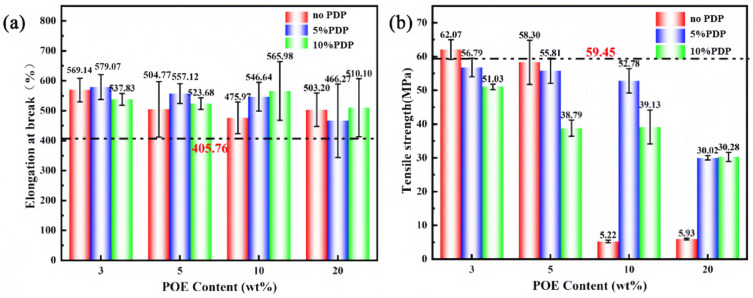

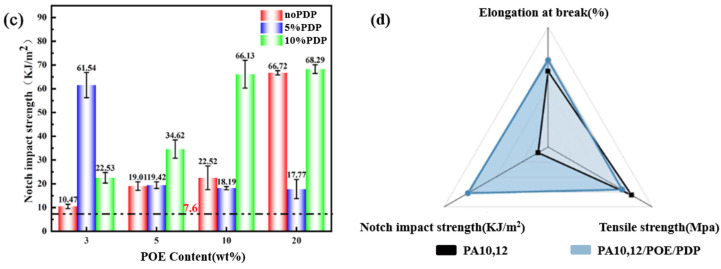
(**a**) Elongation at break of PA10,12 and PA10,12/POE/PDP composites. (**b**) Tensile strength of PA10,12 and PA10,12/POE/PDP composites. (**c**) Notch impact strength of PA10,12 and PA10,12/POE/PDP composites. (**d**) Mechanical properties of PA10,12, PA10,12/POE and PA10,12/POE/PDP composites.

The mechanical properties of all the composites, as characterized by their elongation at break and tensile strength, are graphically represented in Figure 10. For the PA10,12/POE binary composite system, it was observed that both the elongation at break and tensile strength of the composites decreased with an increase in the POE content. Noted that the composite with an addition of 3% POE achieved the optimal mechanical properties, with a maximum elongation at break of 569% and a tensile strength of 62.07 MPa, respectively. This finding suggests that while the incorporation of POE can improve the flexibility of the PA10,12 matrix, excessive amounts may lead to a compromise in the mechanical integrity of the composite. However, the tensile strength dramatically decreased to 5.22 MPa and notched impact strength dropped to 10.47 MPa with further increasing POE content to 10 wt%, attributed to the incompatible nature between PA matrix and POE elastomer. According to previous research [16], PDP molecules could significantly enhance PA impact strength, but it would decrease composite tensile strength at the same time. From Figure 10, one can observe that tensile strength of ternary composite decreased obviously when incorporation of 10% PDP. Interestingly, the introduction of 5% PDP into the PA10,12/POE system resulted in a simultaneously enhancement of both tensile strength and notched impact strength. When the PDP content reaching 5%, the elongation at break slightly increased compared with binary PA/POE composite. The notch impact strength of PA/POE/PDP 92/3/5 exhibit an excellent notched impact strength of 61.5 kJ/m^2^, as shown in Figure 10c, which is an impressive eightfold increase over that of the neat PA system. Concurrently, the tensile strength of these composites achieved a commendable 56.8 MPa, a value nearly equivalent to the tensile strength of the neat PA system. Consequently, the ternary composite realized a significant improvement in impact strength without any detriment to its tensile properties, indicating a synergistic reinforcement and toughening effect on the PA composite, as further illustrated in Figure 10d.

**Figure 11:**
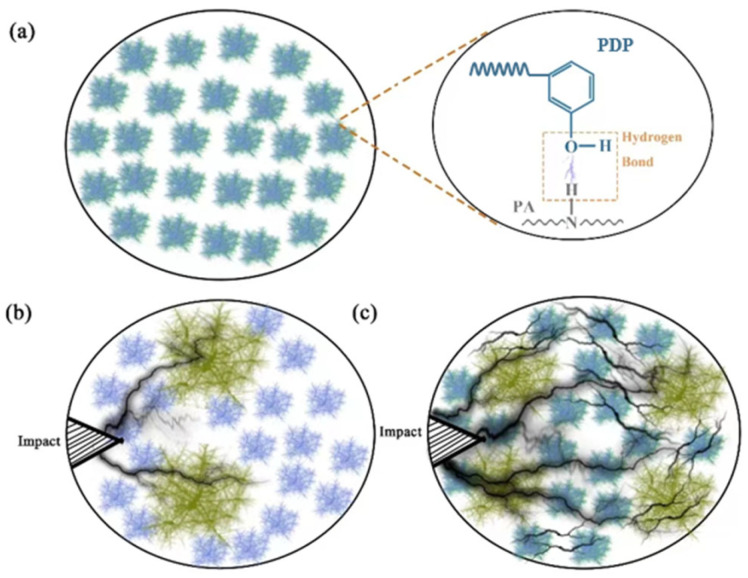
The schematic picture for (**a**) well-dispersed POE and intermolecular H-bonds between PA and PDP; (**b**) impact imposing on the binary PA/POE composite; (**c**) impact imposing on the trinary PA/POE/PDP composite.

In the binary PA10,12/POE composite system, the dispersed POE soft phase could weaken the external impact force by attenuating a large amount of energy to form numerous silver stripes and shear bands. Moreover, the soft POE phase would deform in response to external force, thereby enhancing the toughness of the matrix (Figure 11b) by depleting external energy [28]. Consequently, the tensile strength would dramatically decrease due to the low modulus of POE. On the other hand, as a result of the H-bonds between PDP and the PA matrix (Figure 11a), the PDP molecules could act as compatibilizers to effectively disperse POE elastomers in the matrix, as observed in SEM images (Figure 2). Moreover, the POE phase decreased, and their interface gradually became blurred due to improved compatibility between two phases, facilitating stress transfer between PA10,12 matrix and dispersed POE phase. Consequently, the introduction of PDP as a compatibilizer in the ternary PA/POE/PDP composite leads to the formation of a multitude of micro-cracks. These micro-cracks serve as energy dissipation sites, effectively absorbing and distributing the impact energy (Figure 11c). This energy dissipation mechanism significantly enhances the impact strength performance of the composite, while simultaneously preserving its tensile strength. The compatibilizing effect of PDP promotes a more uniform distribution of stress within the composite, reducing the likelihood of crack propagation and enhancing the overall mechanical integrity of the material. The mechanical properties of PA10,12, PA10,12/POE, and PA10,12/POE/PDP composites are depicted in Figure S3.

## 4. Conclusions

In summary, this study presents a straightforward melt blending technique to enhance the toughness and strength of PA10,12 through the establishment of intermolecular hydrogen bond interactions. We integrate the broad peaks and shifts in absorption wavenumbers from the infrared spectrum, the reduction in melting point from the DSC curves, and the decrease in crystallization rate from the XRD curves to comprehensively demonstrate the formation of hydrogen bonds. The interface interaction between PA10,12 and POE was investigated using SEM imaging. Additionally, the crystallization properties and kinetics of the composites were characterized using X-ray diffraction (XRD) and differential scanning calorimetry (DSC) techniques. Notably, the crystallization temperature (*T_c_*) decreased from 169.1 °C to 161.1 °C upon the addition of 5% PDP and 3% POE. The analysis of crystallization kinetics revealed that the semi-crystallization time *t*_1/2_ and F(T) values of the PA10,12/POE/PDP ternary composite increased. This suggests that the addition of POE and PDP reduced the crystallization rate of PA10,12 and prolonged the crystallization time. Furthermore, mechanical testing demonstrated a significant enhancement in the impact strength of the ternary PA10,12/POE/PDP composite, which increased from 8 kJ/m^2^ to 61.54 kJ/m^2^, marking a 720% improvement over the neat PA10,12. Moreover, the elongation at break was significantly improved, reaching up to 579%. Collectively, this research introduces a novel toughening strategy to enhance the toughness of PA composites while maintaining their stiffness, achieved through the strategic formation of intermolecular hydrogen bonds.

## Figures and Tables

**Table 1:** The nomination of PA10,12 composites.

Samples	PA10,12/w%	POE/w%	PDP/w%
PA10,12	100	0	0
PA10,12/POE (97/3)	97	0	3
PA10,12/POE (95/5)	95	0	5
PA10,12/POE (90/10)	90	0	10
PA10,12/POE (80/20)	80	0	20
PA10,12/POE/PDP (92/3/5)	92	3	5
PA10,12/POE/PDP (90/5/5)	90	5	5
PA10,12/POE/PDP (85/10/5)	85	10	5
PA10,12/POE/PDP (75/20/5)	75	20	5
PA10,12/POE/PDP (87/3/10)	87	3	10
PA10,12/POE/PDP (85/5/10)	85	5	10
PA10,12/POE/PDP (80/10/10)	80	10	10
PA10,12/POE/PDP (70/20/10)	70	20	10

**Table 2:** Crystallization and melting parameters of PA10,12, PA10,12/POE and PA10,12/POE/PDP composites.

Samples PA10,12/POE/PDP	*T_c_* (°C)	*T_oneset_* (°C)	*T_oneset_* − *T_c_*	*T_m_* (°C)	*X_c_*/%
100/0/0	166. 5	169.0	2.5	192.3	18.9
97/3/0	163.9	166.6	2.7	190.6	18.6
95/5/0	163.2	165.8	2.6	189.4	36.4
90/10/0	165.8	168.4	2.6	191.4	21.0
80/20/0	165.4	167.7	2.3	189.6	19.0
92/3/5	161.1	164.4	3.3	189.4	15.6
90/5/5	160.1	163.7	3.6	187.8	28.6
85/10/5	157.7	161.7	4.0	185.8	22.9
75/20/5	158.6	162.8	4.2	187.3	24.9
87/3/10	158.6	162.6	4.0	188.3	16.7
85/5/10	160.2	164.1	3.9	188.5	26.4
80/10/10	157.3	161.3	4.0	185.5	25.9
70/20/10	158.6	162.3	3.7	187.2	32.4

**Table 3:** Isothermal kinetic parameters of PA10,12 and PA10,12/POE/PDP composites at different temperatures.

Samples: PA10,12/POE/PDP	*T* (°C)	*n*	*K* (min^−1^)	*t*_½_ (min)
100/0/0	182	1.9	0.79	0.15
	183	1.9	0.69	0.15
	184	1.9	0.61	0.14
	185	1.9	0.66	0.15
	186	1.9	0.65	0.15
97/3/0	182	2.5	0.98	0.31
	183	2.3	0.85	0.38
	184	2.4	0.96	0.26
	185	2.4	0.96	0.31
	186	2.3	0.83	0.34
92/3/5	182	2.3	1.23	0.40
	183	2.2	1.09	0.40
	184	2.3	1.22	0.38
	185	2.3	1.24	0.37
	186	2.3	1.19	0.36
87/3/10	182	2.4	1.45	0.39
	183	2.3	1.22	0.38
	184	2.4	1.31	0.38
	185	2.4	1.31	0.31
	186	2.3	1.21	0.35

**Table 4:** The parameters of non-isothermal crystallization kinetics can be ascertained through the application of the *Mo* equation.

Samples	*X_c_* (%)	20	40	60	80
PA10,12	a	1.33	1.40	1.51	1.59
	F(T)	0.44	0.73	0.90	1.16
PA10,12/POE	a	1.92	2.04	2.13	2.23
	F(T)	0.09	0.49	0.77	1.14
PA10,12/POE/5%PDP	a	1.91	1.97	2.03	2.14
	F(T)	0.51	0.94	1.27	1.58
PA10,12/POE/10%PDP	a	2.18	2.14	2.14	2.16
	F(T)	0.41	1.01	1.41	1.78

## Data Availability

Data are contained within the article and Supplementary Materials.

## Supplementary Materials

The following supporting information can be downloaded at: https://www.mdpi.com/article/10.3390/polym16131915/s1, Figure S1: (a) XRD photos of PA10,12/POE. (b) XRD photos of PA10,12/POE/5%PDP composites. (c) XRD photos of PA10,12/POE/10%PDP composites.; Figure S2: (a) Non isothermal crystallization curves of PA10,12. (b) Non isothermal crystallization curves of PA10,12/POE. (c) Non isothermal crystallization curves of PA10,12/POE/5%PDP composites. (d) Non isothermal crystallization curves of PA10,12/POE/10%PDP composites.; Figure S3: (a) Elongation at break of PA10,12/POE composites. (b) Notch impact strength of PA10,12/POE composites. (c) Tensile strength of PA10,12/POE composites. (d) Elongation at break of PA10,12/POE/PDP composites. (e) Notch impact strength of PA10,12/POE/PDP composites. (f) Tensile strength of PA10,12/POE/PDP composites.
