# Supplementary material for: 3-Pentadecylphenol (PDP) as a Novel Compatibilizer for Simultaneous Toughened and Reinforced PA10,12 Composites
Source: Polymers (Basel). 2024 Jul 4;16(13):1915. doi: 10.3390/polym16131915 (PMC11243882; doi:10.3390/polym16131915)
Supplement: Supplementary file 1 [file polymers-16-01915-s001.zip › polymers-3026586-supplementary.pdf]

Supporting information

3-pentadecylphenol (PDP) as a Novel Compatibilizer for Simultaneous Toughened and Reinforced PA10,12/POE/PDP Composites

Yuwei Jin[1,2,3], Qi Zhang[4], Xiaokun Zhai[1], Hao Teng[1], Youmei Du[1], Jing Lu[1], Sumaiya Farzana [5], Patrick C. Lee[5], Ruiyan Zhang[4,], Faliang Luo[1,2,*]*

[1] *State Key Laboratory of High-efficiency Utilization of Coal and Green Chemical Engineering, Ningxia University, Yinchuan 750021, China*

[2] *School of Chemistry and Chemical Engineering, Ningxia University, Yinchuan, 750021, China*

[3] *Chuanghe New Material Technology Jiangsu Co., Ltd, Yangzhou 225000, China*

[4] *Key Laboratory of Photovoltaic Materials, School of Materials and New Energy, Ningxia University, Yinchuan, 750021, China*

[5] *Multifunctional Composites Manufacturing Laboratory (MCML), Department of Mechanical and Industrial Engineering, University of Toronto, Toronto M5S 3G8, Canada*

* Corresponding author: Ruiyan Zhang (*ruiyan.zhang@nxu.edu.cn*).Faliang Luo (*flluo@iccas.ac.cn*),

In order to explore the effects of PDP and POE on the crystal form of PA10,12, XRD patterns of PA10,12 and composites were observed by X-ray diffraction, as shown in Figure S1. It can be clearly seen from Figure S1 (a) that the position of the characteristic diffraction peak of the composite material does not change significantly after the addition of POE, but the diffraction peak decreases, which indicates that the addition of POE does not produce new crystal forms of PA10,12. This is consistent with the results of DSC, and is consistent with the behavior of melting enthalpy

reduction. It can also be seen from Figure S1 (b) (c) that the change law of the characteristic diffraction peak is consistent with that of Figure S1 (a), showing a trend of first increasing and then decreasing. When POE content is 10%, the characteristic diffraction peak of the composite material reaches the maximum, possibly because the increase of POE content changes the degree of crystal imperfection of the composite material.

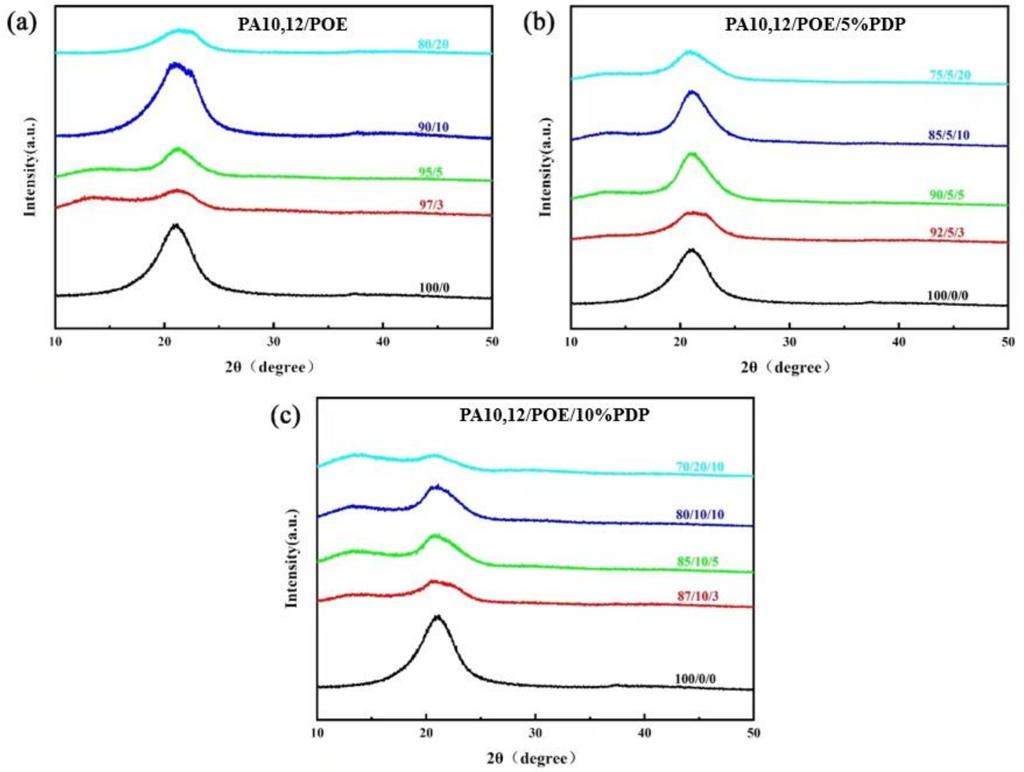

Figure S1 (a)XRD photos of PA10,12/POE. (b)XRD photos of PA10,12/POE/5%PDP composites. (c)XRD photos of PA10,12/POE/10%PDP composites.

As can be seen from Figure S2, with the increase of crystallization rate, the initial crystallization temperature and crystallization peak temperature of PA10,12, PA10,12/POE, PA10,12/POE/PDP composites both deviated to the low temperature direction with the increase of crystallization rate. This is because when the cooling rate is low, the activity capacity of the molecular chain is strong, and the molecular chain has sufficient time to

arrange. Therefore, larger crystallization rates occur at higher temperatures. When the cooling rate increases, the molecular chain has no time to disrupt the rearrangement, so crystallization occurs at lower temperatures. Therefore, with the increase of cooling rate, the regularity of molecular chains in the arrangement of composite materials becomes worse and worse. During melting, PA10,12, POE and PDP molecular chains are diffused, moved and entangled. In the cooling process, the molecular chain of PA10,12 is bound by the POE molecular chain, which hinders its crystal growth, and the chip perfection of the composite material is not as good as that of pure PA10,12.

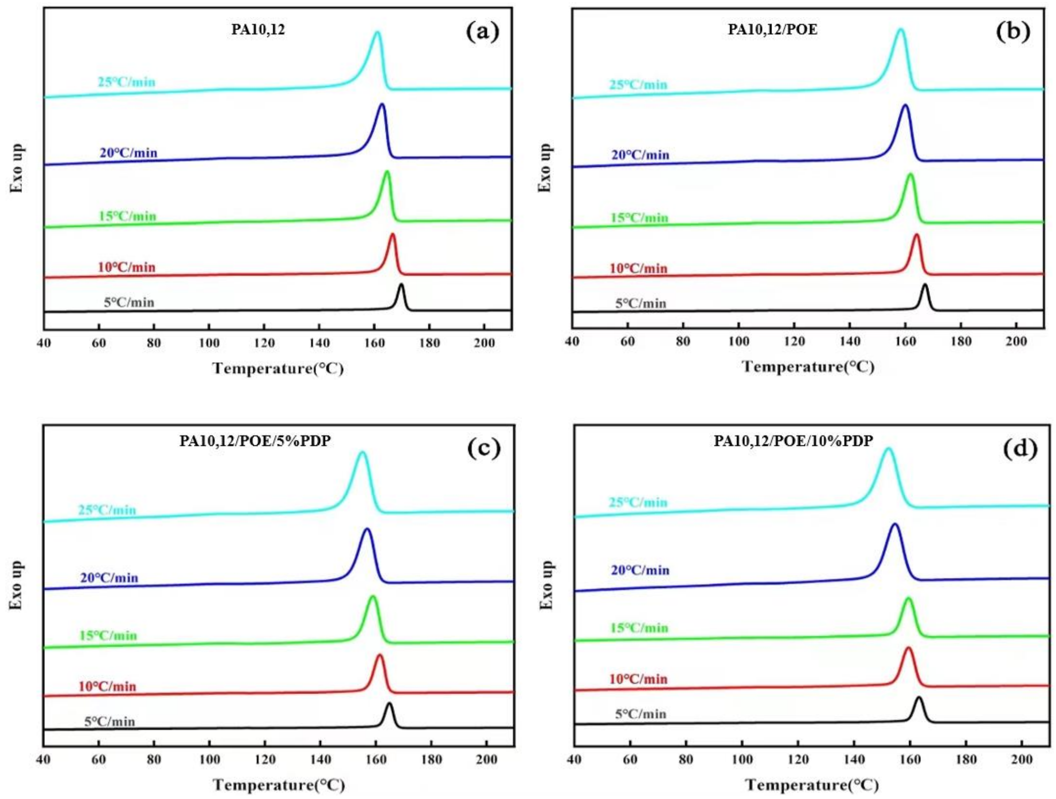

Figure S2 (a)Non isothermal crystallization curves of PA10,12. (b)Non isothermal crystallization curves of PA10,12/POE. (c)Non isothermal crystallization curves of PA10,12/POE/5%PDP composites. (d)Non isothermal crystallization curves of PA10,12/POE/10%PDP composites.

The mechanical properties of the composite are shown in Figure S3. The

elongation at break and tensile strength of the composite both increase first and then decrease with the increase of POE content. The notch impact strength increases with the increase of POE content, and when POE content is 3%, the elongation at break and tensile strength of the composite are optimal. They are 569% and 62.07 MPa respectively, while the notch impact strength at this time is only 10.47 KPa, which is due to the fact that PA10,12 and POE belong to incompatible systems and are caused by large interface tension. However, with the addition of PDP, the tensile strength and notch impact strength of the composite are improved. When the PDP content is 5% and the POE content is 3%, the composite achieves the optimal comprehensive performance, and the elongation at break of the ternary composite can reach 579% (Figure S3 (d)), and the tensile property is almost no loss (Figure S3 (f)). The most outstanding advantage compared with the binary PA10,12/POE composites is that the terpolymer system has an excellent notched impact strength of 61.54 kJ/m^2 (Figure S3 (e)), which is 8 times higher than that of pure PA10,12. This is because in the composite material, the POE content is low, and under the action of PDP, POE can be relatively evenly dispersed in PA10,12, when the external force is acted on, POE can be used as a stress concentration point to disperse the external force on the sphere.

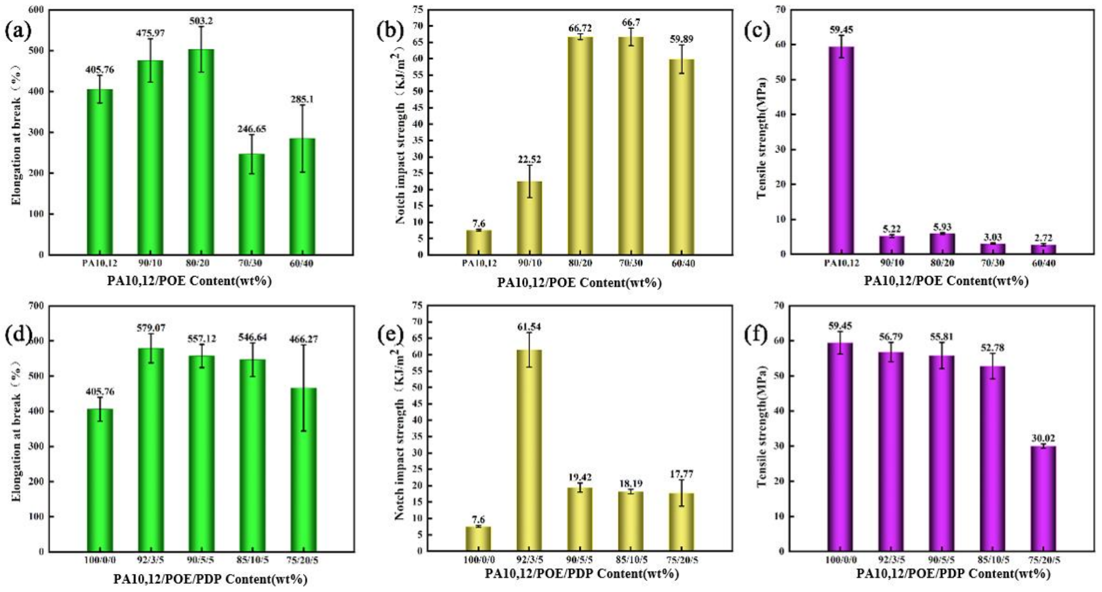

Figure S3 (a) Elongation at break of PA10,12/POE composites. (b) Notch impact strength of PA10,12/POE composites. (c) Tensile strength of PA10,12/POE composites. (d) Elongation at break of PA10,12/POE/PDP composites. (e) Notch impact strength of PA10,12/POE/PDP composites. (f) Tensile strength of PA10,12/POE/PDP composites.
